# Indication-Specific Adverse Events and Temporal Trends of Lumen-Apposing Metal Stents: A National Postmarketing Surveillance Analysis (2020–2025)

**DOI:** 10.1055/a-2979-6046

**Published:** 2026-10-21

**Authors:** Ishaan Vohra, Muhammad K. Hasan, Harishankar Gopakumar, Dennis Yang, Deepanshu Jain, Natalie Cosgrove, Maham Hayat, Ranya Ahmed, Abdul Mohammed, Zubair Khan, Carlos-Rodriguez Bertran, Mustafa A. Arain

**Affiliations:** 1 Gastroenterology 17120 University of Illinois Chicago College of Medicine at PeoriaPeoriaIllinoisUnited States; 2 Center for Interventional Endoscopy 440172 AdventHealth OrlandoOrlandoFloridaUnited States; 3 Gastroenterology 1861 Brigham and Women’s HospitalBostonMassachusettsUnited States; 4 Medicine 6243 University of Central FloridaOrlandoFloridaUnited States

**Keywords:** lumen-apposing metal stent, gastroenterostomy, adverse events, postmarketing surveillance, patient safety

## Abstract

**Background and Aims** The AXIOS stent is a lumen-apposing metal stent widely used for endoscopic ultrasound-guided drainage of pancreatic fluid collections, biliary drainage, and gastroenterostomy. We analyzed device problems and patient adverse events reported to the FDA Manufacturer and User Facility Device Experience (MAUDE) database (2020–2025) with indication-specific risk stratification.

**Methods** We performed a retrospective analysis of 1775 AXIOS adverse event reports from the FDA MAUDE database, systematically classified by procedural indication, device problem, and patient outcome. Each report was assigned to a single highest-severity category, and within-indication proportions were calculated.

**Results** Annual reports increased by 232%, from 144 in 2020 to 478 in 2025. The indications comprised pancreatic drainage (46.2%), biliary drainage (35.7%), and gastroenterostomy (9.5%). Device problems included positioning/malpositioning (59.0%), distal flange activation failure (19.4%), migration (7.3%), and proximal detachment (4.5%). Gastroenterostomy carried the highest within-indication proportions of mortality (11.8%), perforation (6.5%), and infection (4.1%), exceeding biliary (3.9%, 6.3%, 1.4%) and pancreatic drainage (2.3%, 3.7%, 1.5%).

**Conclusions** AXIOS adverse event reporting increased by 232% from 2020 to 2025, reflecting expanding utilization and enhanced surveillance. Gastroenterostomy carried the highest within-indication burden of serious adverse events. The marked rise in distal flange activation failures—coinciding with December 2025 manufacturer field safety notice—demonstrates postmarketing surveillance value and underscores the need for indication-specific risk stratification and prospective registries capturing operator- and device-level detail that passive surveillance cannot provide.

## Introduction

Endoscopic ultrasound (EUS)-guided transmural interventions have fundamentally altered therapeutic approaches for pancreatic-biliary diseases, including pancreatic fluid collections, biliary obstruction, gastric obstruction, and gastroenteric access. 1 2 Central to this revolution is the development of lumen-apposing metal stents (LAMS), which facilitate creation and maintenance of transmural anastomoses between luminal structures. 3 4 The AXIOS stent system (Boston Scientific Corporation, Marlborough, MA), introduced in 2011–2012, represents the first and most widely adopted LAMS device, distinguished by its bilateral anchor flange design and electrocautery-enhanced delivery system. 5 6 7

AXIOS stents have demonstrated clinical efficacy across multiple indications. For pancreatic walled-off necrosis (WOPN) and pseudocyst drainage, EUS-guided drainage has supplanted surgical and percutaneous approaches, offering comparable efficacy with reduced morbidity and shorter hospital stays, with LAMS offering ease of deployment and a larger lumen for drainage compared to plastic stents. 8 9 In malignant gastric outlet obstruction, EUS-guided gastroenterostomy (EUS-GE) provides faster oral intake resumption and reduced reintervention rates compared with enteral stenting. 10 11 For malignant biliary obstruction, EUS-guided choledochoduodenostomy has emerged as an effective alternative when endoscopic retrograde cholangiopancreatography (ERCP) fails, with comparable technical success and stent patency. 12 13 In addition, EUS-guided gallbladder drainage offers a minimally invasive approach for definitive gallbladder drainage in nonoperative surgical candidates. 6

Despite widespread adoption, comprehensive safety data remain fragmented. Published studies predominantly consist of single-center series or meta-analyses with heterogeneous outcome reporting and variable follow-up durations. 9 14 15 16 Adverse events, including bleeding, perforation, stent migration, and infection, have been documented, with reported rates varying widely based on indication, operator experience, and procedural technique. 9 17 18 19 20 21 Critically, most studies lack sufficient power to detect indication-specific risk profiles or temporal trends in device performance as utilization expands beyond expert centers.

The FDA Manufacturer and User Facility Device Experience (MAUDE) database provides a unique opportunity to examine real-world safety signals through postmarket surveillance. 22 Previous MAUDE analyses have identified important safety patterns for LAMS devices, including bleeding, migration, and deployment-related adverse events, although comprehensive AXIOS-specific analyses stratified by indication and temporal trends remain limited. 23 Furthermore, rapid expansion of LAMS indications and increasing operator experience necessitate temporal trend analysis to distinguish evolving practice patterns from inherent device-related risks. 24 25 26 27 Understanding indication-specific complication profiles is essential for evidence-based clinical decision-making, informed consent, and risk mitigation strategies. We hypothesized that adverse event patterns and complication rates would vary significantly across procedural indications and that temporal trends would reveal evolving safety profiles as clinical experience matured. This study systematically analyzed 1775 AXIOS adverse event reports from the FDA MAUDE database (2020–2025) to characterize indication-specific risks, temporal trends, device-related problems, and patient outcomes in contemporary practice.

## Methods

### Data Source and Extraction

We conducted a comprehensive analysis of adverse events associated with AXIOS stents using the FDA MAUDE database, a publicly accessible passive surveillance system used to collect medical device adverse event reports from manufacturers, healthcare facilities, and healthcare professionals. 28 MAUDE database files spanning January 2020 through December 2025 were systematically queried, and all reports listing “AXIOS” in the brand name field were extracted and compiled. The study window was defined a priori to encompass a minimum of five complete calendar years of MAUDE reporting, a duration sufficient to distinguish sustained temporal trends from year-to-year variation in a passive surveillance system; all complete annual files available at the time of query were included, yielding a six-year window spanning the period of broad LAMS adoption across all major procedural indications.

The initial compiled dataset contained 1811 records. Thirty-five rows representing MAUDE database disclaimer and footer text rather than adverse event reports were removed, yielding 1776 reports. Following duplicate removal based on FDA Report Number (1 duplicate identified), 1775 unique adverse event reports remained. All reports were included in the analysis to maximize comprehensiveness, including 108 events with documented event dates prior to 2020 that were reported within the 2020–2025 database files. For temporal trend analyses examining year-over-year changes, we focused on the 1667 events with event dates from January 1, 2020 to December 31, 2025. Event dates in the 2025 dataset extended through December 10, 2025, representing approximately 94% of the calendar year. Two further checks were performed. Report-number structure was examined for MAUDE follow-up (supplemental) reports; all identifiers conformed to either the standard manufacturer registrant–year–sequence format or the voluntary MedWatch format, with no supplemental suffix present, and no collapsing of index and follow-up reports was required. Records sharing byte-identical narrative text under distinct report numbers were also screened; 10 such pairs were identified and individually reviewed.

### Event Classification

Each adverse event report underwent systematic classification by analyzing multiple data fields including Event Text (narrative description), Device Problem (manufacturer-coded categories), and Patient Problem (clinical outcome codes). A standardized algorithmic approach was developed and uniformly applied to all reports.

Procedural indication was classified through keyword detection in event narratives as: (1) pancreatic pseudocyst or WOPN drainage (keywords: “pseudocyst,” “WOPN,” “walled-off necrosis,” “pancreatic fluid collection,” “necrosectomy”); (2) gastroenterostomy (keywords: “gastroenterostomy,” “gastric outlet obstruction,” “GOO,” “duodenal obstruction”). This category encompasses EUS-GE for gastric outlet obstruction and EUS-directed transgastric ERCP (EDGE) procedures where an LAMS is used to facilitate gastric remnant access in a patient with a Roux-en-Y gastric bypass; (3) biliary drainage, encompassing both common bile duct drainage (choledochoduodenostomy, hepatogastrostomy) and gallbladder drainage (cholecystostomy) (keywords: “choledochoduodenostomy,” “hepatogastrostomy,” “biliary drainage,” “bile duct,” “cholangiocarcinoma,” “gallbladder,” “cholecystitis,” “cholecystostomy”); or (4) not specified when procedural context was ambiguous or absent.

Procedural approach (transgastric vs. transduodenal) was assessed by screening narratives for explicit anatomical terms. However, approach was inconsistently documented and frequently ambiguous: many narratives referenced both terms or neither, and approach was strongly confounded with procedural indication. Approach-specific outcome analysis was therefore not pursued ( **Supplementary Fig. 1** [available in online version only]). Stent type was categorized as Hot AXIOS (electrocautery-enhanced delivery), Cold AXIOS (standard delivery), or type not specified. Stent size (diameter × length in millimeters) was extracted when explicitly documented; however, size was recorded in only a minority of reports, precluding size-stratified outcome analysis.

Device Problems were classified using both the standardized Device Problem field and free-text Event Text analysis. Categories included: positioning/malpositioning problems, distal flange activation failures, proximal flange detachment issues, stent migration/dislodgement, material integrity problems, premature deployment, breakage, and difficulty with stent removal. Distal flange activation failures were identified by the keywords “Activation Failure” or “Activation Problem,” representing technical problems with electrocautery-enabled distal flange deployment. Proximal flange detachment issues were identified by the keyword “Detachment,” representing premature release or separation of the proximal anchor flange.

Patient outcomes were determined through systematic keyword searching of Event Text and Patient Problem fields. Outcomes classified included: death (mortality was identified through Event Text analysis using death-related keywords: “death,” “died,” “deceased,” “fatal,” “mortality,” “passed away,” explicitly excluding “expired” which consistently referred to device expiration dates rather than patient mortality); bleeding/hemorrhage; perforation; and infection/sepsis/peritonitis. Events coded as “No Clinical Signs, Symptoms, or Conditions” without other adverse outcomes were classified as device malfunctions without patient harm. Multiple outcomes could be coded for individual events. Each report was assigned to a single, highest-severity adverse event category (mortality > perforation > bleeding > infection > migration), so categories are mutually exclusive; within-indication proportions therefore reflect the report’s primary adverse event. All classifications were performed using the standardized algorithm. To assess classification accuracy, a 10% random sample ( *n* = 178) underwent independent review against the algorithm output, demonstrating high concordance for procedural indication and patient mortality.

### Statistical Analysis

Descriptive statistics were calculated as frequencies and percentages for categorical variables. Temporal trends were assessed by stratifying the 1667 events occurring during 2020–2025 by calendar year. Year-over-year growth rates were calculated as the percentage change from the preceding year. Complication rates (mortality, bleeding, perforation, infection, migration) were calculated as proportions of total events within each procedural indication category, using the complete 1775-event dataset. Given the passive surveillance nature of MAUDE and the absence of denominator data (total AXIOS procedures performed), true procedure-based incidence rates could not be calculated. 29 30 Therefore, all reported rates represent proportions of adverse events within the analyzed cohort rather than population-based complication rates. 22 31 Comparative analyses between indications were descriptive without formal statistical testing, as MAUDE data limitations preclude adjustment for confounding variables including patient selection, disease severity, operator experience, and institutional factors. 22 29 30 32 33 All analyses were performed using Python version 3.11 (Python Software Foundation, Wilmington, DE). Data processing and manipulation were conducted using pandas (version 2.0). 34 Visualizations were generated using matplotlib (version 3.7). Numerical computations were performed using NumPy (version 1.24). 34 This study utilized publicly available deidentified data and did not require institutional review board approval. 35

## Results

### Overall Adverse Event Trends and Temporal Patterns

The analysis identified 1775 unique AXIOS adverse event reports submitted to the FDA MAUDE database during 2020–2025 ( Table 1 ). Of these, 1667 had event dates within the study window, while the remaining 108 (6.1%) had event dates prior to 2020 but were reported during the study period. Annual reports increased from 144 events in 2020 to 478 events in 2025—an overall 232% increase ( Fig. 1 )—with the steepest year-over-year growth occurring between 2023 and 2024 (242–411 events, +69.8%). Event dates in the 2025 dataset extended through December 10, 2025, capturing approximately 94% of the calendar year.

**Table 1:** Overall characteristics of Lumen-Apposing metal stent adverse events ( *n* = 1775).

Characteristic	N	Percentage (%)
Total adverse events	1775	100
Year of event (2020–2025)		
2020	144	8.1
2021	172	9.7
2022	220	12.4
2023	242	13.6
2024	411	23.2
2025*	478	26.9
Events occurring 2020–2025†	1667	93.9
Procedural indication		
Pancreatic/WOPN	820	46.2
Biliary drainage	633	35.7
Gastroenterostomy	169	9.5
Other fluid collection/abscess	21	1.2
Not specified	132	7.4
FDA event classification		
Injury	937	52.8
Malfunction	783	44.1
Death	54	3.0
Not specified	1	0.1

**Fig. 1:**
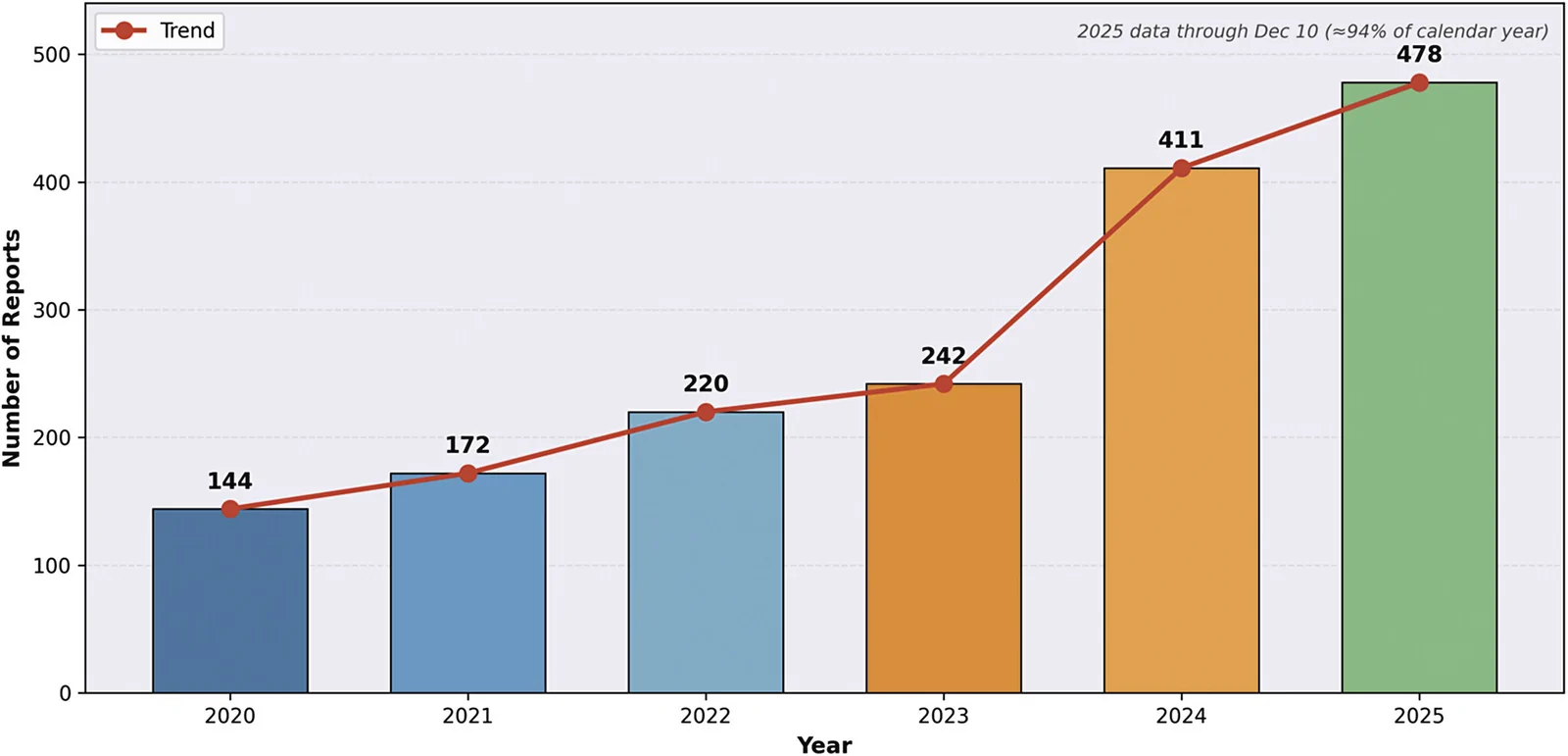
Annual Adverse Event Reports for the Lumen-Apposing Metal Stent System (2020–2025). Bar chart displays annual adverse event counts, demonstrating an approximately 232% increase from 144 reports in 2020 to 478 in 2025. The trend line indicates sustained year-over-year growth across the entire study period. Counts reflect the 1667 reports with event dates during 2020–2025; the 2025 dataset includes events through December 10, 2025, representing approximately 94% of the calendar year. Value labels above each bar indicate exact annual counts.

Events were categorized by the FDA Event Type field as injury (52.8%, *n* = 937), malfunction (44.1%, *n* = 783), or death (3.0%, *n* = 54). A broader mortality definition—encompassing the FDA “Death” event type together with Patient Problem codes for multiple organ failure and cardiac arrest and narrative-confirmed deaths—identified 65 fatal events. The most frequent Patient Problem code was “No Clinical Signs, Symptoms or Conditions,” recorded in 1305 reports (73.5%), indicating that the database is dominated by device-level events without documented clinical sequelae.

### Distribution by Procedural Indication

Pancreatic pseudocyst or walled-off pancreatic necrosis drainage was the most frequently reported indication, accounting for 46.2% ( *n* = 820) of adverse events ( Table 1 ). Biliary drainage—combining EUS-guided malignant biliary drainage and EUS-guided gallbladder drainage—comprised 35.7% ( *n* = 633), and gastroenterostomy for gastric outlet obstruction accounted for 9.5% ( *n* = 169). A further 1.2% ( *n* = 21) of reports involved off-label drainage of other fluid collections or abscesses, and 7.4% ( *n* = 132) lacked sufficient narrative detail to assign a procedural indication, predominantly predeployment or off-table device malfunctions ( Table 1 ). Temporal analysis demonstrated a sustained, uninterrupted year-over-year increase in biliary drainage reports throughout the study period, rising from 28 events in 2020 to 218 in 2025; reports for the other major indications also increased over the period but with less consistent year-to-year trajectories ( Fig. 2 ).

**Fig. 2:**
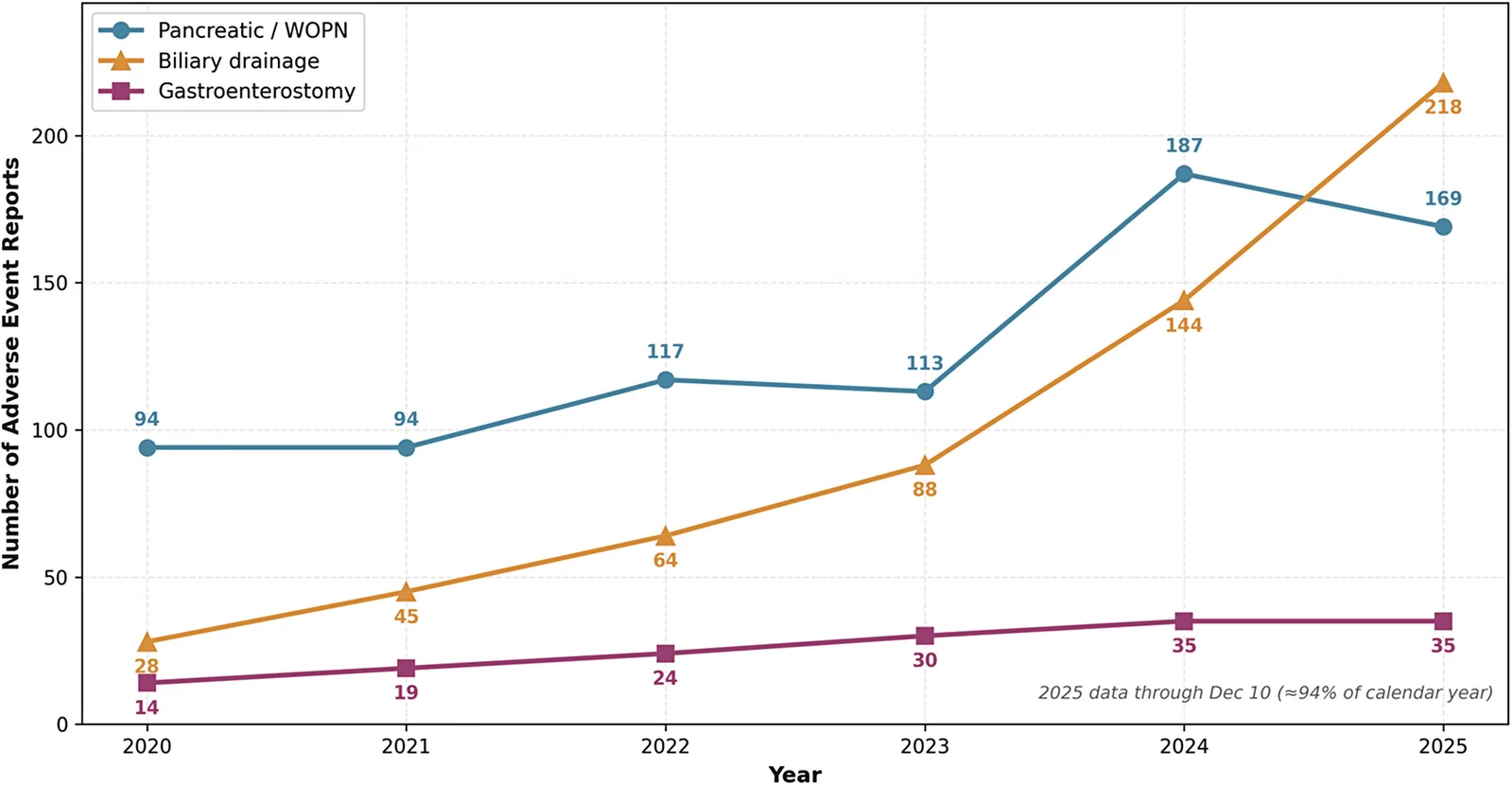
Temporal Trends in Adverse Events by Procedural Indication (2020–2025). Annual adverse event reports for the three principal procedural indications among the 1667 reports with event dates during 2020–2025. Biliary drainage (combining EUS-guided malignant biliary drainage and gallbladder drainage) showed the steepest and most sustained increase, rising from 28 reports in 2020 to 218 in 2025 and surpassing pancreatic drainage in 2025. Pancreatic/WOPN drainage increased from 94 to 169 reports with year-to-year fluctuation, and gastroenterostomy increased modestly from 14 to 35 reports. The 2025 dataset includes events through December 10, 2025 (≈94% of the calendar year). Data labels indicate exact annual counts. Values represent counts of adverse event reports, not event rates; MAUDE provides no procedural denominator, so between-indication differences in trajectory reflect reporting volume and cannot be interpreted as differences in procedural risk.

### Adverse Outcomes by Procedural Indication

The patterns of severe adverse events differed substantially by procedural indication ( Table 2 ). Among the 65 procedure-related mortality reports, biliary drainage accounted for 25 events (38.5%), gastroenterostomy for 20 (30.8%), and pancreatic drainage for 19 (29.2%). Of the 81 reports of procedure-related perforation, biliary drainage contributed 40 (49.4%), pancreatic drainage 30 (37.0%), and gastroenterostomy 11 (13.6%). Stent migration was distributed similarly between biliary drainage (42 events, 44.2%) and pancreatic drainage (40 events, 42.1%), with gastroenterostomy contributing 7 events (7.4%). Bleeding and infectious complications were concentrated in pancreatic and biliary drainage, reflecting their larger report volumes (bleeding: 46 [54.8%] pancreatic drainage and 22 [26.2%] biliary drainage; infection: 12 [40.0%] pancreatic drainage and 9 [30.0%] biliary drainage, respectively).

**Table 2:** Adverse outcomes and complications by procedural indication ( *n* = 1775).

Adverse event	Pancreatic/WOPN‡	Biliary drainage§	GOO/EUS-GE¶	Other fluid collection/abscess	Unknown/not specified	TOTAL
Mortality	19 (2.3%)	25 (3.9%)	20 (11.8%)	0 (0.0%)	1 (0.8%)	65 (3.7%)
Perforation	30 (3.7%)	40 (6.3%)	11 (6.5%)	0 (0.0%)	0 (0.0%)	81 (4.6%)
Bleeding	46 (5.6%)	22 (3.5%)	11 (6.5%)	2 (9.5%)	3 (2.3%)	84 (4.7%)
Infection	12 (1.5%)	9 (1.4%)	7 (4.1%)	1 (4.8%)	1 (0.8%)	30 (1.7%)
Migration	40 (4.9%)	42 (6.6%)	7 (4.1%)	2 (9.5%)	4 (3.0%)	95 (5.4%)
Other AE	673 (82.1%)	495 (78.2%)	113 (66.9%)	16 (76.2%)	123 (93.2%)	1420 (80.0%)
**TOTAL**	**820 (100.0%)**	**633 (100.0%)**	**169 (100.0%)**	**21 (100.0%)**	**132 (100.0%)**	**1775 (100.0%)**

Expressed as a proportion of reports within each indication, gastroenterostomy ( *n* = 169) carried the highest burden of serious primary adverse events. Mortality was the primary event in 11.8% of gastroenterostomy reports, compared with 3.9% for biliary drainage and 2.3% for pancreatic drainage; the corresponding figures were 6.5%, 6.3%, and 3.7% for perforation, and 4.1%, 1.4%, and 1.5% for infection. This pattern indicates that although gastroenterostomy represents a small share of total reports, it is disproportionately weighted toward the most severe outcomes.

### Patient Adverse Events

Patient adverse events demonstrated distinct patterns and temporal trends ( Figs. 3 and 4A ). Hemorrhage and bleeding represented the most common patient complication, occurring in 7.9% (140/1775) of reports ( Fig. 4A ); annual counts increased from 11 events in 2020 to a peak of 26 in 2024 ( Fig. 3 ). Perforation occurred in 5.3% (94/1775) of reports, increasing from 5 events in 2020 to 23 in 2025—an approximately fourfold rise over the study period ( Fig. 3 ). Pain was documented in 3.4% (60/1775) of reports, fever in 1.7% (30/1775), and sepsis or septic shock in 1.2% (22/1775) ( Fig. 4A ).

**Fig. 3:**
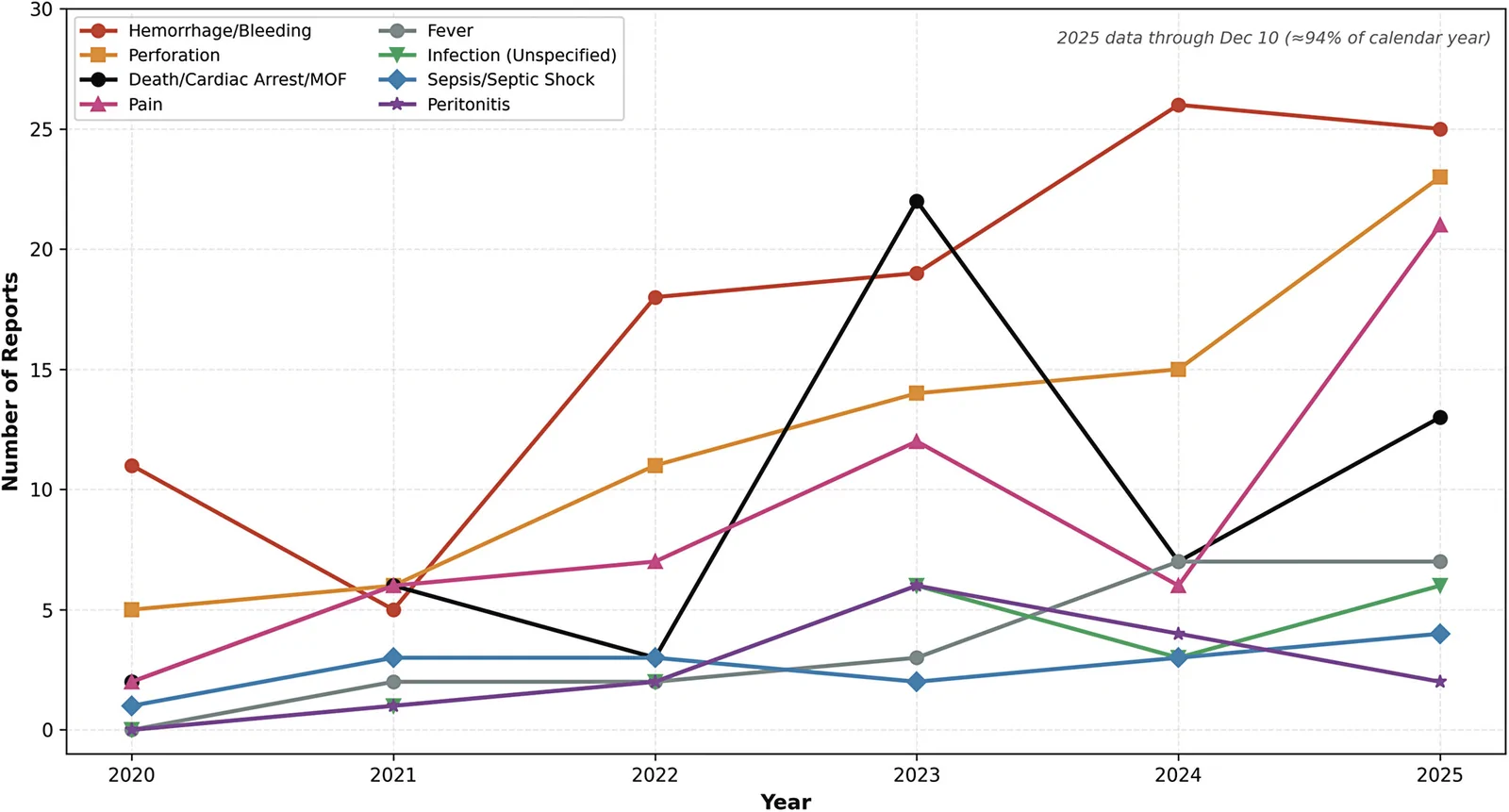
Patient Adverse Event Trends by Year (2020–2025). Multiline graph showing the temporal evolution of eight patient adverse event categories among reports submitted during the study period. Hemorrhage/bleeding was the most frequently reported event in most years, rising from 11 reports in 2020 to a peak of 26 in 2024. Perforation increased overall from 5 reports in 2020 to 23 in 2025. Death/cardiac arrest/multiple organ failure showed a variable year-to-year pattern, including a peak of 22 reports in 2023. Pain, fever, infection, sepsis, and peritonitis were reported at lower frequencies throughout. The 2025 dataset includes events through December 10, 2025 (≈94% of the calendar year). Categories are non-mutually exclusive.

**Fig. 4:**
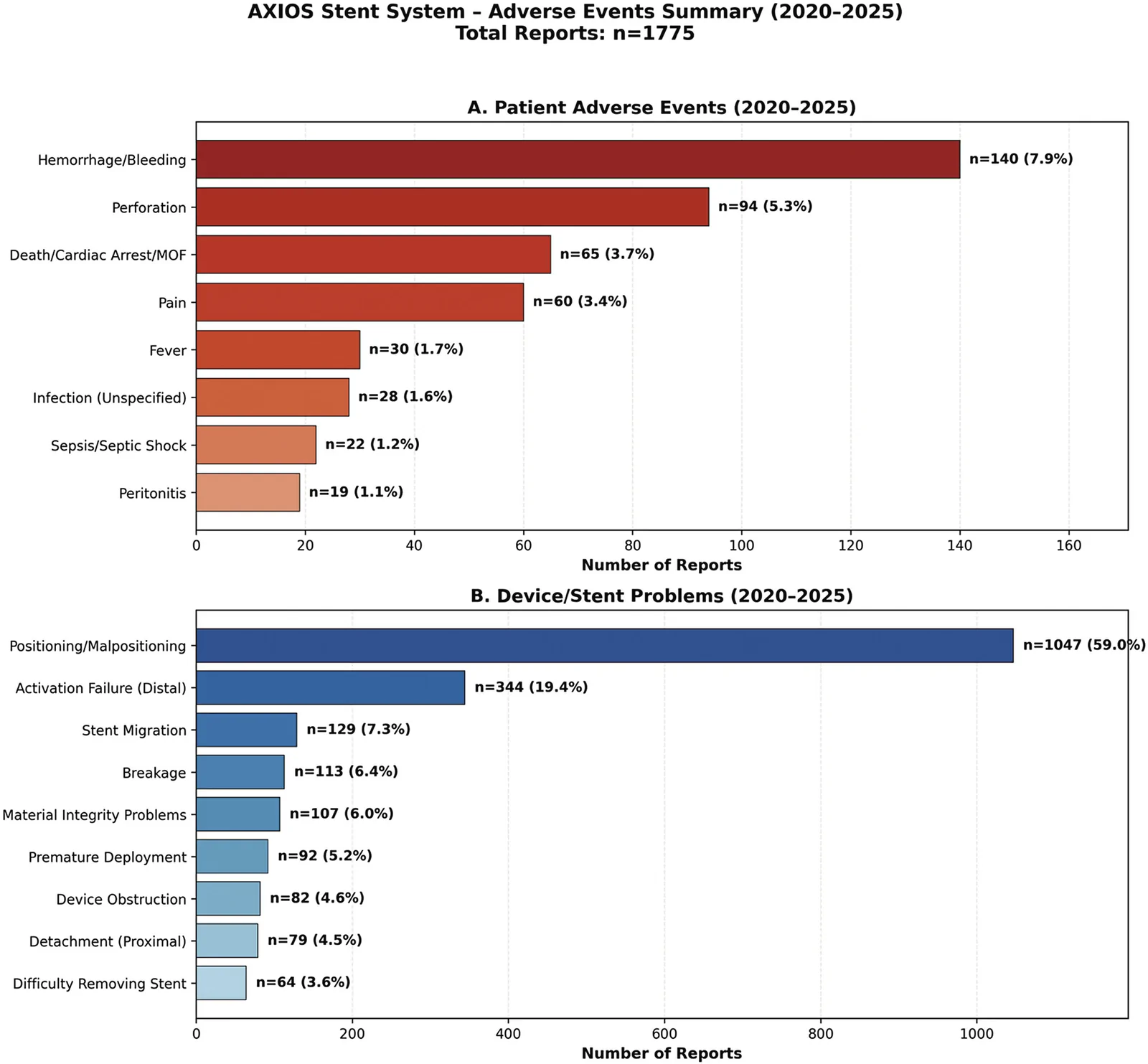
Summary of Patient Adverse Events and Device Problems (2020–2025). Horizontal bar charts displaying ranked frequencies of ( **A** ) eight patient adverse event categories and ( **B** ) nine device/stent problem categories, with exact counts and percentages of total reports ( *n* = 1775). Among patient events, hemorrhage/bleeding was most frequent (7.9%), followed by perforation (5.3%). Among device problems, positioning/malpositioning predominated (59.0%), and distal flange activation failures (19.4%) substantially exceeded proximal flange detachment (4.5%). Categories are non-mutually exclusive; a single report may contribute to more than one category.

Death or cardiac arrest was explicitly recorded in the Patient Problem field in only 0.2% (3/1775) of reports—far lower than the 3.7% (65/1775) mortality identified through comprehensive narrative text and multifield analysis. This discrepancy reflects incomplete Patient Problem field completion and underscores the importance of structured narrative review in MAUDE-based adverse event surveillance.

### Device-Related Problems

Positioning and malpositioning issues represented the predominant device problem, occurring in 59.0% (1047/1775) of reports ( Fig. 4B ). This composite category combined the MAUDE codes Positioning Problem ( *n* = 424), Activation, Positioning or Separation Problem ( *n* = 401), Positioning Failure ( *n* = 256), and Difficult or Delayed Positioning ( *n* = 76), reflecting difficulty achieving appropriate stent position, inadequate flange apposition, and suboptimal anastomotic alignment. Distal flange activation failures (MAUDE code Activation Failure) were the second most common device problem, affecting 19.4% (344/1775) of reports ( Fig. 4B ) and representing failure of the electrocautery system to deploy the distal flange during initial deployment. This category showed the most pronounced temporal escalation of any device problem, rising approximately 11-fold from 13 reports in 2020 to 145 in 2025 ( Fig. 5 ).

**Fig. 5:**
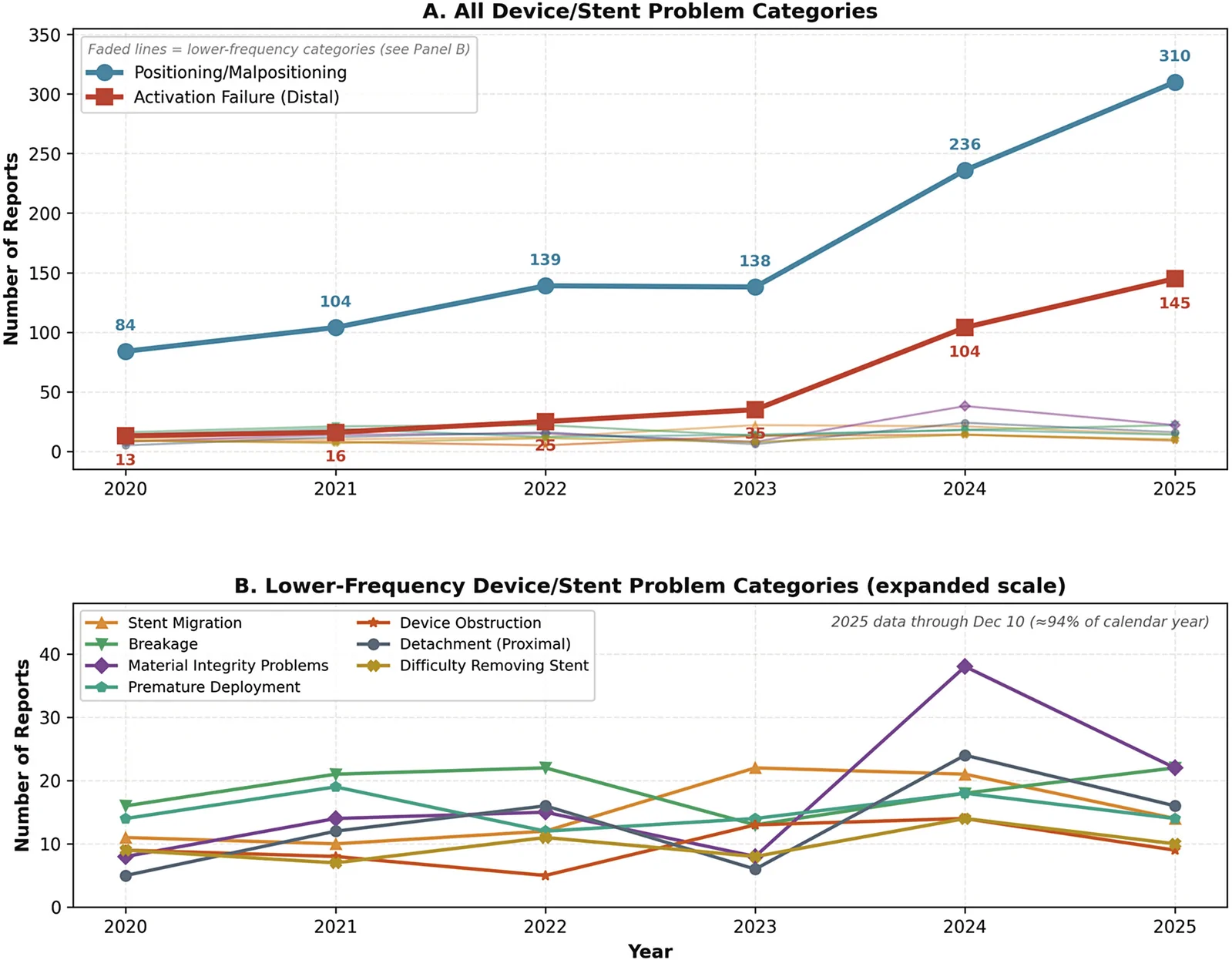
Device/Stent Problem Trends by Year (2020–2025). ( **A** ) Annual frequencies of all 9 device problem categories; positioning/malpositioning (84–310 reports) and distal flange activation failures (13–145 reports, an approximately 11-fold increase) are shown as emphasized lines, with the seven lower-frequency categories displayed faded for context. ( **B** ) The same seven lower-frequency categories shown on an expanded scale for legibility. Proximal flange detachment remained substantially lower than distal activation failure throughout, consistent with proximal flange deployment occurring under direct endoscopic visualization. The 2025 dataset includes events through December 10, 2025 (≈94% of the calendar year). Categories are non-mutually exclusive.

Proximal flange detachment (MAUDE code Detachment of Device or Device Component) occurred in 4.5% (79/1775) of reports ( Fig. 4B ), encompassing premature proximal flange release, detachment during or after deployment, and inadequate proximal apposition. Distal flange activation failures were thus approximately four times more frequent than proximal flange detachment, a disparity consistent with the greater technical complexity of distal flange deployment, which occurs without direct endoscopic visualization. Stent migration was documented as a device problem in 7.3% (129/1775) of reports, with annual counts ranging from 10 to 22 and no clear monotonic trend ( Figs. 4B and 5 ). Material integrity problems—combining the MAUDE codes Material Integrity Problem ( *n* = 54), Material Deformation ( *n* = 44), and Material Separation ( *n* = 12)—occurred in 6.0% (107/1775) of reports, whereas stent breakage was documented in 6.4% (113/1775) and premature activation in 5.2% (92/1775) ( Figs. 4B and 5 ).

### Stent Characteristics and Data Quality

Stent size was the most prominent data-quality gap. MAUDE adverse event reports do not include a structured device-size field, so stent dimensions were recoverable only from free-text narrative descriptions. Explicit stent dimensions were documented in just 236 of 1775 reports (13.3%); the remaining 1539 (86.7%) contained no extractable size information. This severe under-documentation precluded meaningful size-stratified outcome analysis and represents an important limitation of MAUDE for postmarket surveillance of devices, such as AXIOS, that are marketed in multiple dimensional configurations.

## Discussion

This analysis of 1775 AXIOS adverse event reports submitted to MAUDE between 2020 and 2025 produced three findings that matter for practice. Annual reporting more than tripled, the risk profile differed sharply by procedural indication, and reports of distal flange activation failure increased roughly 11-fold—a climb that coincided, almost to the month, with a manufacturer recall. Taken together, these observations show both what passive surveillance can do and where it falls short.

The rise in reports is best understood as wider use of the device combined with better reporting, not as a device that is becoming less safe. AXIOS use has expanded well beyond drainage of pancreatic fluid collections into biliary, gallbladder, and gastroenteric applications, and report volume has followed that expansion. 24 25 36 Because MAUDE records events but not the number of procedures performed, this growth reflects changing practice rather than changing risk per procedure.

The indications also diverge in their reporting trajectories, and this divergence warrants care in interpretation. Biliary drainage reports increased without interruption across the study period, whereas gastroenterostomy reports remained comparatively flat. Because the figures display report counts rather than rates and the MAUDE database provides no procedural denominator, this contrast cannot be attributed to differences in safety. The most parsimonious explanation is differential growth in procedural volume: biliary and gallbladder drainage have expanded most rapidly of the three indications and have diffused well beyond tertiary centers, consistent with a rapidly enlarging denominator, while EUS-GE remains a lower-volume procedure concentrated in fewer high-volume centers and accounted for only 9.5% of reports. A stable count against a slowly growing denominator is compatible with either a stable or a falling event rate, and the two cannot be distinguished here. The stability should not, however, be read as reassurance: gastroenterostomy carries the highest within-indication proportion of mortality, perforation, and infection in the cohort, so the flat trajectory reflects report volume rather than severity.

The differences between indications are the most useful finding here, and they have a clear mechanistic explanation. Measured as a proportion of reports within each indication, gastroenterostomy carried the heaviest burden of serious events—mortality 11.8%, perforation 6.5%, and infection 4.1%—well above biliary drainage (3.9%, 6.3%, 1.4%) and pancreatic drainage (2.3%, 3.7%, 1.5%). The gradient makes intuitive sense: a misdeployed stent at a free gastroenteric anastomosis spills enteric contents into the peritoneum, whereas a misdeployed stent during pancreatic drainage decompresses into a contained, walled-off collection. The same logic explains why gastroenterostomy, despite making up only a small share of reports, repeatedly emerges as the highest-acuity indication. The one exception is migration, which was most frequent not in gastroenterostomy but in biliary drainage (6.6%)—likely the result of peristaltic shear at the cholecystoenteric and choledochoenteric anastomoses and weaker tissue ingrowth across noninflamed walls. Two established measures—larger-diameter stents and prophylactic fixation with endoscopic suturing or over-the-scope clips—have reduced migration in comparative studies and deserve indication-specific consideration. 37 38 39 40

These proportions should not be read as procedural complication rates. MAUDE overrepresents severe outcomes and lacks a procedure-volume denominator, so absolute figures are best read as proportions of reported events rather than procedural risk. Reported perforation proportions for gastroenterostomy illustrate this: the nonexclusive estimate (10.7%) closely matches a prior MAUDE analysis (10.4%) and exceeds prospective series (2–5%), 23 whereas the primary-event estimate used here (6.5%) is lower by virtue of the mutually exclusive classification. The two methods differ in magnitude but not in direction—across all surveillance modes, gastroenterostomy emerges as the highest-acuity indication, and that ranking, rather than any single rate, should guide practice.

On the device itself, the defining signal is the contrast between the two anchor flanges. Distal flange activation failures (19.4%) outnumbered proximal detachment (4.5%) by roughly four to one, a difference that follows directly from how the device deploys. The distal flange is released blindly through tissue, leaving it exposed to electrocautery malfunction, poor apposition, and premature activation, while the proximal flange deploys under direct endoscopic vision. 3 27 41 42 43 More telling is the trajectory: distal activation failures increased from 13 reports in 2020 to 145 in 2025, an 11-fold increase that far outpaced the roughly threefold growth in overall reporting. In December 2025, Boston Scientific issued an urgent field safety notice for specific AXIOS configurations, citing deployment failures. 41 That an independently derived MAUDE signal converged with manufacturer action is the clearest evidence in this dataset of what surveillance can accomplish.

A second safety consideration warrants mention. AXIOS is FDA approved for transgastric or transduodenal drainage of pancreatic pseudocysts and walled-off necrosis, and for EUS-guided gallbladder drainage in nonoperative surgical candidates; its use for EUS-GE in gastric outlet obstruction remains off-label. In parallel, the dual-balloon enteroclysis catheter (NAJA, Chess Medical, Montreal, Canada) has been adopted to facilitate EUS-GE by distending an isolated small-bowel segment prior to LAMS deployment, and early experience suggests the technique may reduce procedural adverse events through improved target visualization and stable apposition. 44 It should be noted, however, that NAJA is FDA-cleared for endoscopic evaluation of the small intestine, not for the creation of enteric anastomoses, so its use in EUS-GE is itself off-label. These dual off-label considerations underscore the importance of careful patient selection, transparent informed consent, and continued development of dedicated devices and approval pathways for non-PFC LAMS indications.

These findings carry several practical implications. Patients considered for gastroenterostomy should be counseled explicitly about elevated mortality and perforation risk, and surgical gastrojejunostomy or enteral stenting should be genuinely weighed when life expectancy and oncologic context allow. Patients undergoing biliary drainage should be counseled specifically about migration. Pancreatic drainage carries the most favorable mortality profile of the three, with bleeding as its main procedural concern. For endoscopists, distal flange deployment is the critical step—the largest category of device failure and the fastest growing. One factor this dataset cannot measure, but that almost certainly shapes these numbers, is operator experience. Successful LAMS deployment depends on advanced EUS skill, and part of the rise in reported complications may simply reflect the technique spreading beyond high-volume expert centers into less experienced hands. MAUDE records the event but never the endoscopist, so this influence is invisible in the data even as it likely contributes to it. As LAMS use continues to diffuse into broader practice, structured proctorship—together with defined competency thresholds and standardized curricula—is likely to matter more than didactic instruction alone, although the present data cannot evaluate this directly. A recent survey of advanced endoscopy fellowship programs documented marked variability in LAMS training exposure and competency assessment, supporting the case for more standardized curricula as these techniques continue to disseminate. 45

The strengths of this study are its scale and detail: the largest AXIOS-specific MAUDE cohort reported to date, six continuous years of data, and indication-level profiling drawn from systematic review of report narratives. Its limitations are inherent to MAUDE. Passive surveillance captures only a small and biased fraction of true events, cannot establish causality, and provides no procedure denominator. 22 46 47 Structured data fields were often uninformative—“No Clinical Signs” filled 73.5% of the Patient Problem field, and stent dimensions could be recovered from narrative text in only 13% of reports—which required labor-intensive manual review and ruled out size-stratified analysis. The rise in reports also blends genuine safety signals with growth in use and improved reporting, and reporting lag right-censors the most recent years: 10–23% of events from 2020 to 2024 were not logged until a later calendar year, so 2025 counts are necessarily incomplete and the temporal increases reported here are conservative estimates ( **Supplementary Fig. 2** [available in online version only]). These constraints define what the analysis can claim: relative, hypothesis-generating signals, not population incidence. Three further limitations warrant mention. First, the biliary drainage category pools EUS-guided drainage of the biliary tree with EUS-guided gallbladder drainage. These are distinct procedures with different access windows and plausibly different risk profiles, and separating them would be clinically valuable, but we were unable to do so reliably: gallbladder-related terms appear in approximately one quarter of narratives, yet in the majority of these, the only mention lies within the manufacturer’s standard Directions-for-Use text, which enumerates all cleared indications irrespective of the procedure performed. Keyword-based assignment would therefore have produced substantial misclassification. Second, MAUDE does not permit determination of the interval from LAMS placement to death: the database records individual adverse event reports rather than a followed cohort, patients are neither censored nor tracked longitudinally after a reported event, and no procedure-date field exists, so temporal attribution of mortality to the index procedure is not possible. Third, 10 pairs of reports shared identical narrative text under distinct report numbers; most reflect established MAUDE conventions, namely separate reports filed for each device used in a single procedure and literature-derived reports sharing a common source narrative, but because manufacturer narratives are templated a small residue of true duplication cannot be excluded. At fewer than 1% of records, any such residue is too small to alter the reported proportions.

In summary, AXIOS adverse events show distinct, indication-specific patterns. Gastroenterostomy is the highest-acuity indication for mortality, perforation, and infection; biliary drainage carries the greatest migration burden; pancreatic drainage is comparatively the safest. The 11-fold rise in distal flange activation failures, confirmed by a concurrent manufacturer recall, shows that MAUDE—for all its limitations—can detect clinically meaningful device signals before they are formally characterized. Confirming and quantifying these signals will require prospective multicenter registries with complete case ascertainment, standardized outcome definitions, and mandatory device characterization—the steps needed to turn the relative signals reported here into the absolute, denominator-based risk estimates that truly evidence-based device selection requires. 46 47 48 49 50 51 52 53 Such registries should record the target organ explicitly, permitting the separation of gallbladder from biliary tree drainage that passive surveillance narratives cannot support, and should record the date of index placement, permitting attribution of mortality to a defined postprocedural interval. Finally, because the December 2025 field safety notice postdates this data window, its effect on reporting patterns could not be assessed; reinterrogation of MAUDE after a sufficient postnotice interval would permit direct evaluation of whether the recall altered the frequency or character of reported deployment failures.

## Key Messages

1. Among LAMS indications, gastroenterostomy carries the highest within-indication burden of serious adverse events—mortality (11.8%), perforation (6.5%), and infection (4.1%)—substantially exceeding biliary and pancreatic drainage, underscoring the need for careful patient selection and explicit preprocedure counseling.
2. Distal flange activation failures (19.4%) were reported approximately four times more often than proximal flange detachment (4.5%), reflecting the technical difficulty of deploying the distal flange without direct endoscopic visualization, with implications for operator training and device refinement.
3. MAUDE surveillance can detect real-world safety signals: distal flange activation failures increased roughly 11-fold over the study period, a trend that coincided with a December 2025 Boston Scientific field safety notice citing deployment failures.
4. Operator experience is not captured in MAUDE and could not be assessed here; whether the approximately 232% rise in adverse event reporting between 2020 and 2025 partly reflects diffusion of these techniques beyond expert centers is a hypothesis this dataset can generate but cannot test.

## Data Availability

The data analyzed in this study are publicly available from the US FDA MAUDE database ( https://www.accessdata.fda.gov/scripts/cdrh/cfdocs/cfmaude/search.cfm ). Derived analytic datasets are available from the corresponding author upon reasonable request.

## Declaration of GenAI Use

Generative artificial intelligence (Claude, Anthropic) was used in the preparation of this manuscript to assist with data processing, analysis code development, and drafting and language editing of the text. Figures and tables are data visualizations derived from statistical analysis using standard visualization libraries; no generative AI was used to create images. All analytic outputs and findings were reviewed and verified by the authors, and all interpretations and conclusions are those of the authors. The authors take full responsibility for the integrity of the data, the accuracy of the analysis, and the content of this manuscript.

## Supplementary Material

Ergänzendes Material Supplementary Material
