# Supplementary material for: Indication-Specific Adverse Events and Temporal Trends of Lumen-Apposing Metal Stents: A National Postmarketing Surveillance Analysis (2020–2025)
Source: Endosc Int Open. 2026 Oct 21;14:a29796046. doi: 10.1055/a-2979-6046 (PMC13758975; doi:10.1055/a-2979-6046)

Figure S1: Procedural approach documentation in the dataset (n=1,775). (A) Explicit mention of transgastric or transduodenal access in report narratives. Only 731 reports (41.2%) specified a single unambiguous approach; 490 (27.6%) referenced both terms and 554 (31.2%) referenced neither. (B) Approach documentation stratified by procedural indication, illustrating strong confounding between approach and indication. Because of this ambiguity and confounding, approach-specific outcome analysis was not performed.

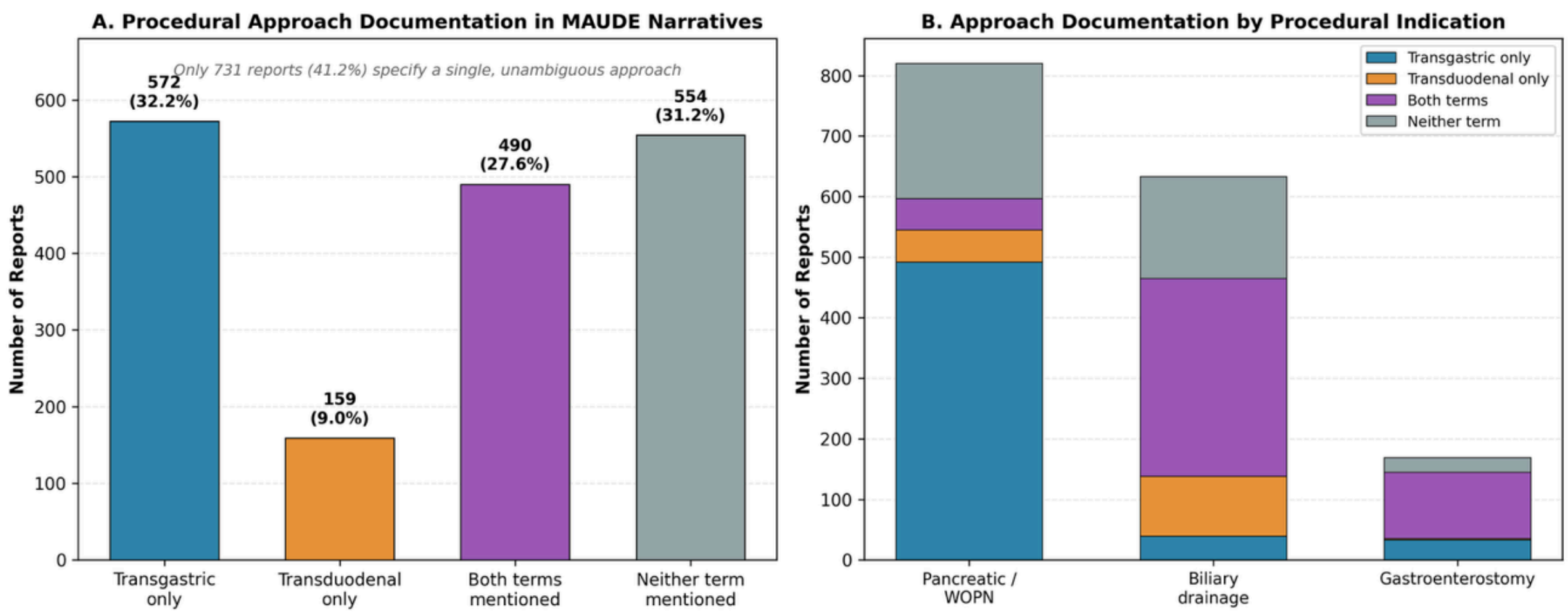

Figure S2: Reporting lag and right-censoring in the dataset (n=1,775). (A) Distribution of the interval between event date and FDA receipt. The median lag was 26 days, but the distribution had a pronounced right tail; 6.6% of reports were received more than two years after the event (long tail truncated at 730 days for display; maximum 17.3 years). (B) For each event year, reports received within the same calendar year versus a later calendar year. Between 10% and 23% of events from 2020–2024 were reported in a subsequent year. Because late reports for 2025 events have not yet had time to accrue, 2025 counts are necessarily incomplete, and observed temporal trends for the most recent year represent conservative estimates.

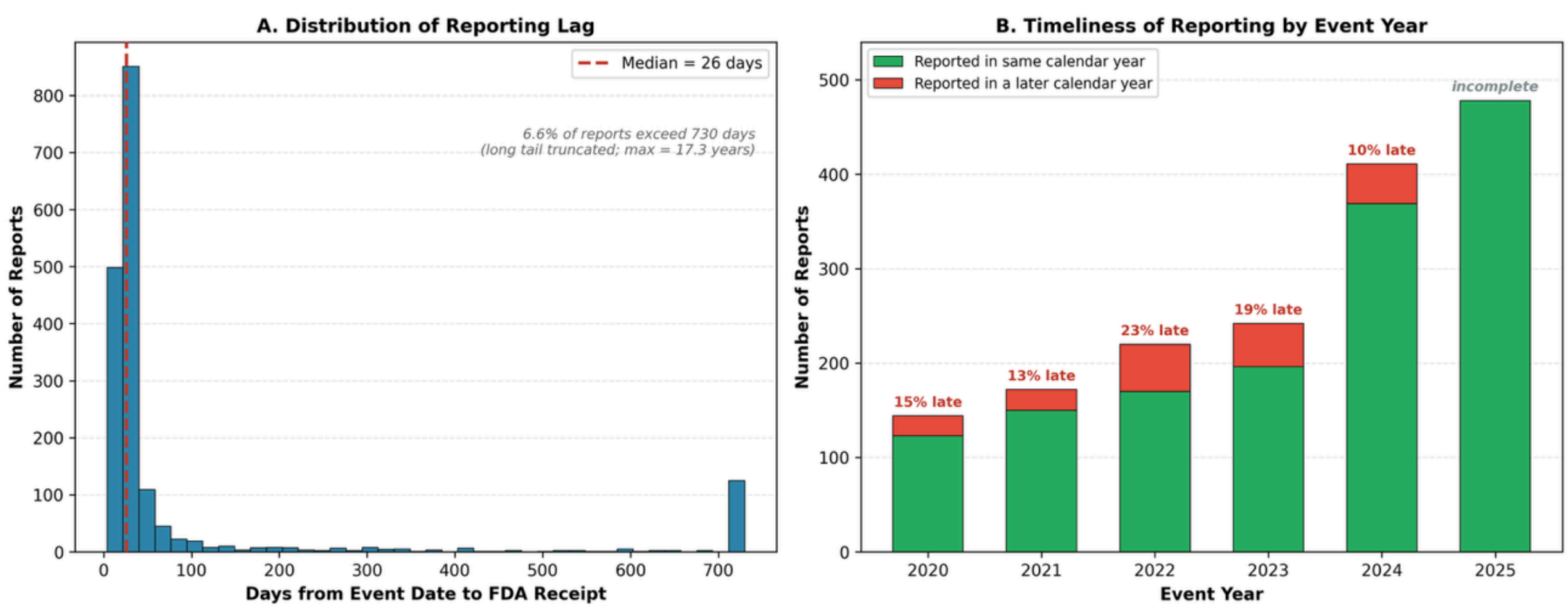

Supplement: Supplementary file 1 [file 10-1055-a-2979-6046_29803970.pdf]
